# Preventive antimicrobial action and tissue architecture ameliorations of *Bacillus subtilis* in challenged broilers

**DOI:** 10.14202/vetworld.2021.523-536

**Published:** 2021-02-26

**Authors:** Essam S. Soliman, Rania T. Hamad, Mona S. Abdallah

**Affiliations:** 1 Department of Animal Hygiene, Zoonosis, and Animal Behavior, Faculty of Veterinary Medicine, Suez Canal University, Ismailia 41522, Egypt; 2 Department of Pathology, Faculty of Veterinary Medicine, Menoufia University, Al Minufya 33511, Egypt; 3 Department of Avian and Rabbit Medicine, Faculty of Veterinary Medicine, Suez Canal University, Ismailia 41522, Egypt

**Keywords:** broiler chickens, histopathological photomicrographs, immunity, *in vitro* antimicrobial, *in vivo* preventive, probiotics

## Abstract

**Background and Aim:**

Probiotics improve intestinal balance through bacterial antagonism and competitive exclusion. This study aimed to investigate the *in vitr*o antimicrobial activity, as well as the *in viv*o preventive, immunological, productive, and histopathological modifications produced by probiotic *Bacillus subtili*s.

**Materials and Methods:**

The *in vitro* antimicrobial activities of *B. subtilis* (5×10^6^ CFU/g; 0.5, 1.0*, 1.5, and 2.0 g/L) were tested against *Escherichia coli* O157: H7, *Salmonella* Typhimurium, *Candida albicans*, and *Trichophyton mentagrophytes* after exposure times of 0.25, 0.5, 1, and 2 h using minimal inhibitory concentration procedures. A total of 320 1-day-old female Ross broiler chickens were divided into five groups. Four out of the five groups were supplemented with 0.5, 1.0*, 1.5, and 2.0 g/L probiotic *B. subtilis* from the age of 1 day old. Supplemented 14-day-old broiler chickens were challenged with only *E. coli* O157: H7 (4.5×10^12^ CFU/mL) and *S*. Typhimurium (1.2×10^7^ CFU/mL). A total of 2461 samples (256 microbial-probiotic mixtures, 315 sera, 315 duodenal swabs, and 1575 organs) were collected.

**Results:**

The *in vitro* results revealed highly significant (p<0.001) killing rates at all-time points in 2.0 g/L *B. subtilis*: 99.9%, 90.0%, 95.6%, and 98.8% against *E. coli*, *S*. Typhimurium, *C. albicans*, and *T. mentagrophytes*, respectively. Broilers supplemented with 1.5 and 2.0 g/L *B. subtilis* revealed highly significant increases (p<0.01) in body weights, weight gains, carcass weights, edible organs’ weights, immune organs’ weights, biochemical profile, and immunoglobulin concentrations, as well as highly significant declines (p<0.01) in total bacterial, *Enterobacteriaceae*, and *Salmonella* counts. Histopathological photomicrographs revealed pronounced improvements and near-normal pictures of the livers and hearts of broilers with lymphoid hyperplasia in the bursa of Fabricius, thymus, and spleen after supplementation with 2.0 g/L *B. subtilis*.

**Conclusion:**

The studies revealed that 1.5-2.0 g of probiotic *B. subtilis* at a concentration of 5×10^6^ CFU/g/L water was able to improve performance, enhance immunity, and tissue architecture, and produce direct antimicrobial actions.

## Introduction

Expansion in the poultry industry in the past 50 years has been accompanied by the emergence of a large variety of pathogens and increased microbial resistance. These effects have been attributed to the extensive and abnormal use of antibiotics as prophylactics and therapeutic agents. Recent research has focused on finding alternative supplements that minimize and/or prevent the maintenance of microbial agents in the environment, localization in target tissues, and the production of disease, as well as enhancing immunity levels in broiler chickens [1]. Alternatives such as cytokines, bacteriophages [2], *Nigella sativa* Linn [3], cinnamon (*Cinnamomum zeylanicum*) oil [4], inorganic nano-selenium [5], and probiotics [6] have been suggested and found to be effective in improving performance and immunity.

Probiotics are living microorganisms that, when supplemented in adequate amounts and concentrations, result in health benefits [7]. Probiotic supplementation contributes to beneficial effects, such as improved performance, increased feed efficiency, improved nutrient digestion, and absorption [8], increased egg production, improved health, and reduced pathogenic enzyme secretion [9]. One study that investigated the expanded use of probiotics in poultry farming showed that newly hatched chickens from supplemented flocks could be protected against colonization of *Salmonella* Enteritidis with a dosing suspension of gut contents derived from healthy adult chickens [10], a concept is known as “competitive exclusion”.

In addition to competitive exclusion activity, probiotics exert their effects by regulating intestinal permeability, performing a foster action for the degradation and damage caused by enteric pathogens, enhancing humoral and cellular immunity, and altering agent pathogenicity [11]. Probiotics possess antimicrobial actions against many microorganisms, including *Clostridium perfringens*, *Salmonella* Typhimurium, *Escherichia coli*, and *Staphylococcus aureus* [12], by producing organic acids and antibacterial substances such as hydrogen peroxide, defensins, and bacteriocins.

This study aims to evaluate the *in vitro* antimicrobial action of probiotic *Bacillus subtilis* (5×10^6^ CFU/g) at different concentrations (0.5, 1.0*, 1.5, and 2.0 g/L) against *E. coli* O157: H7 (4.5×10^12^ CFU/mL), *S*. Typhimurium (1.2×10^7^ CFU/mL), *Candida albicans* (2.5×10^6^ CFU/mL), and *Trichophyton mentagrophytes* (2.5×10^6^ CFU/mL) after different exposure intervals (0.25, 0.5, 1, and 2 h) using minimal inhibitory concentration tests. The study also aims to study the *in vivo* preventive and prophylactic actions of probiotic *B. subtilis* on productive performance, histopathological picture, biochemical profile, intestinal microbial load, immune and edible organs’ weights, and immunoglobulin (Ig) concentrations in 14-day-old broiler chickens challenged with *E. coli* O157: H7 (4.5×10^12^ CFU/mL) and *S*. Typhimurium (1.2×10^7^ CFU/mL).

## Materials and Methods

### Ethical approval

The protocol and used materials of the current scientific research were approved by the Scientific Research Ethics Committee, Faculty of Veterinary Medicine, Suez Canal University, Ismailia, Egypt with approval number (2019004).

### Study period and location

The *in-vitro* study was carried out during June and July 2019 in Animal, Poultry and Environmental Hygiene laboratories, Department of Animal Hygiene, Zoonosis and Animal Behavior and Management, Faculty of Veterinary Medicine, Suez Canal University, Ismailia. The *in-vivo* study was conducted from September 5^th^, 2019 to October 12^th^, 2019 in the Broiler Experimental Units, Faculty of Veterinary Medicine, Suez Canal University, Ismailia.

Carcasses’ and organs’ weights, bacteriological, and performance indices assessments were conducted in Animal, Poultry and Environmental Hygiene laboratories, Ismailia. Biochemical and immunoglobulin assays were conducted in the Clinical Pathology Laboratories, Suez Canal University Hospital. Histopathological examinations and photomicrography were conducted in the Pathology Department, Al Minufya.

### *In vitro* antimicrobial action of probiotics

#### Preparation of probiotic suspensions

*B. subtilis* (5×10^6^ CFU/g) powder was purchased from a veterinary pharmacy in Ismailia, Egypt. The bag was opened with care, and four quantities were weighed (0.5, 1.0*, 1.5, and 2.0 g), and each weight was dissolved in 1 L of deionized water to produce the four targeted concentrations: 0.5, 1.0*, 1.5, and 2.0 g/L.

#### Preparation of bacterial and fungal cultures

*E. coli* O157: H7 suspension (1.8×10^7^ CFU/mL) and *S*. Typhimurium lyophilized vials (2.4×10^3^ CFU) were purchased from the Animal Health Research Institute, Dokki, Cairo. *E. coli* O157: H7 was propagated in MacConkey broth (Thermo Scientific™ Oxoid™ MacConkey Broth, CM0505, 500 g) at 44°C for 24 h, while *S*. Typhimurium was propagated in tetrathionate broth (Thermo Scientific™ Oxoid™ Tetrathionate Broth Base, CM0029, 500 g) at 37°C for 24 h, as recommended by Soliman *et al*. [13]. From the positive MacConkey and tetrathionate tubes, 10 μL was dropped onto eosin methylene blue (EMB) agar (EMB, Modified Levine EMB Thermo Scientific™ Oxoid™, CM0069B, 500 g) and CHROMagar™ (BD BBL™ CHROMagar™ *Salmonella* READY-TO-USE Plated Media) and the plates were incubated at 37°C for 24 h [14]. Metallic green colonies of *E. coli* O157: H7 and pink colonies of *S*. Typhimurium were counted, collected, and reconstituted in buffered peptone water (Thermo Scientific™ Oxoid™ Buffered Peptone Water, CM0509B, 500 g), providing suspensions of 4.5×10^12^ and 1.2×10^7^ CFU/mL, respectively.

Suspensions of *C. albicans* and *T. mentagrophytes* (3.5×10^3^ CFU/mL) were provided by the Animal Health Research Institute of Ismailia. Fungal suspensions were propagated in Sabouraud Dextrose Broth (SDB, HIMEDIA^®^ Sabouraud Dextrose Broth, MU033, 500 g) at 37°C for 24 h, dropped onto Sabouraud Dextrose agar (SDA, Thermo Scientific™ Oxoid™ SDA, CM0041, 500 g), and incubated at 37°C for 24 h. Typical colonies were identified by morphological appearance and lactophenol cotton blue stain (Hardy Diagnostics^®^ Lactophenol Cotton Blue, Z68, 15 mL). Colonies were counted, collected, and reconstituted in SDB, providing suspensions of 2.5×10^6^ CFU/mL for each organism.

#### Testing probiotic concentrations against bacterial and fungal cultures

The procedures were carried out using the minimal inhibitory concentration according to Soliman *et al*. [15]. One milliliter from each bacterial or fungal suspension was added to four replicates of 9 mL of each probiotic concentration (0.5, 1.0*, 1.5, and 2.0 g/L), and mixed using a vortexer (Vortex Mixer XH-D, 2800 r/m, 30 W, bowel and disk shapes). After 0.25, 0.5, 1, and 2 h of exposure time, 100 μL were transferred and added to 10-mL physiological saline resuscitation tubes held previously at 4°C and mixed thoroughly by vortexing. The tubes were transferred for the bacteriological assessment.

### *In vivo* efficiency of probiotics

#### Experimental birds: Microclimate and management

A total of 320 1-day-old female Ross chicks were purchased from a company in Ismailia, Egypt. Broiler chicks were divided on their arrival into five groups: G1, G2, G3, G4, and G5 (control), 64 chicks in each group (four replicates of 16 birds). Groups were placed into five independent rooms. The floors of the five rooms were treated with superphosphate (0.5 g/m^2^) before being covered with a hay deep litter system according to Soliman and Hassan [16]. Each room was ventilated through fans on one sidewall and V-shaped windows on the opposing sidewall, contributing to negative pressure cross-ventilation across the room. Broiler chicks in each room were supplied with a continuous lighting regimen using blue LED lights that were adjusted to provide 23 h of lighting and 1 h of darkness a day, as recommended by Soliman and Hassan [17]. The five rooms were secured using the essential biosecurity measures recommended by Soliman and Abdallah [18]: Fly-proof nets, foot dips at the entrance, controlled traffic in and out, restricted access to the rooms, protection of the food storage areas, protection of water resources, and protection from wild bird entrance.

Before the broilers’ arrival, the microclimatic temperature was adjusted and maintained in the five rooms at 35°C (brooding temperature) using halogen heaters (Bravo BR−4T Heater 4 halogen Candles, 2400 W). The indoor temperature was controlled and minimized at a rate of 3°C/week by increasing the ventilation rates during the daylight hours until 26°C was achieved by the end of the 3^rd^ week. Birds were given *ad libitum* access to dechlorinated water and were supplied with a corn-soybean ration to meet their nutritional requirements, as recommended by the National Research Council [19] and Applegate and Angel [20]. Broilers were provided with the corn-soybean ration into two successive stages: The starter ration was provided from 1 to 13 days and constituted 23% protein, 3.81% crude fiber, 4.9% fat, and 2950 kcal/kg energy; and the grower ration was provided from 14 days until the end of the fattening cycle (38 days) and constituted 21% protein, 3.39% crude fiber, 5.8% fat, and 3100 kcal/kg energy. The experiment was designed to last for 38 days. Survival rates, microclimatic thermal level, and humidity level were monitored during the experiment.

Broilers were immunized by mass vaccination in dechlorinated drinking water during the early morning after water deprivation for 2-3 h. Birds were vaccinated against infectious bronchitis using PESTIKAL B1 SPF H120 ≥10^3.5^ live attenuated virus vaccine on the 6^th^ day, against infectious bursal disease using SER-VAC D78 Strain VMG91 ≥10^3.0^ live attenuated vaccine on the 14^th^ and 21^st^ days, and against Newcastle disease (ND) virus using PESTIKAL LaSota ≥10^6.0^ live lentogenic ND virus on the 18^th^ and 28^th^ days.

#### Probiotic supplementation

The chicks in four out of the five broiler groups (G1, G2, G3, and G4) were given drinking water supplemented with *B. subtilis* (5×10^6^ CFU/g) from 1 day of age at a rate of 0.5, 1.0* (recommended by the manufacturer), 1.5, and 2.0 g/L, respectively. The fifth group was used as an unsupplemented control group.

#### E. coli O157: H7 and S. Typhimurium challenge

Broilers in groups G1, G2, G3, and G4 were challenged with *E. coli* O157: H7 (4.5×10^12^ CFU/mL) and *S*. Typhimurium (1.2×10^7^ CFU/mL) in the drinking water at 14 days of age [21].

#### Performance indices

The live body weights of the broiler groups were measured by weighing 56 birds from each group. The number of the weighed birds was calculated using the simple random sampling procedures described by Thrusfield [22] with a 5% error as following:

n=1.96^2^ P_exp_ (1−P_exp_)/d^2^

Where n=required sample size, Pexp=expected prevalence, d=desired absolute precision. Feed intakes expressed by grams (g) were calculated in each group by dividing the total amount consumed by the birds in such a group by the total number of surviving birds in the group. Weight gains expressed by (g), feed conversion ratios (FCR), and performance indices were calculated as recommended by Soliman and Hassan [23].

#### Sampling

A total of 2461 samples, including 256 *in vitro* microbial-probiotic mixtures, 315 sera, 315 duodenal swabs, and 1,575 organs (including bursa of Fabricius, spleen, thymus, heart, and liver) were collected during the period of the study.

Blood samples (a total of 315 sera samples) were obtained by sacrificing 63 birds in each of the five groups by the end of the study (38 days). The blood samples were received in sterile screw-capped centrifuge tubes, held at 37°C for 30 min, and centrifuged (Fisher^®^Thermo Scientific CL10 Centrifuge with F-G3 Rotor, max rpm: 4000) at 3000 rpm for 15 min. Clear non-hemolyzed sera samples were pipetted using an automatic pipette (Thermo Scientific™ Finnpipette™ Adjustable Volume Single-Channel Micro Pipettor, 100-1000 μL volume) into 2-mL Eppendorf tubes and stored at −20°C for the biochemical and immunological assays.

Duodenal swabs (315 swabs and 63 per group) were collected from the intestines of sacrificed broilers’ and placed in 9 mL of buffered peptone water (Thermo Scientific™ Oxoid™ Buffered Peptone Water, CM0509B, 500 g); and the *in vitro* microbial-probiotic mixtures (256 samples, 4 replicates×4 contact times×4 cultures×4 probiotic concentrations) in the physiological saline resuscitation tubes were transferred for bacteriological assessment.

A total of 315 birds were slaughtered after blood sampling and decapitation; the shanks and feet were removed with a knife, and birds were de-feathered and eviscerated of all organs except kidneys. The carcasses were weighed and expressed in grams (carcass weight: CW/g). Edible organs such as the heart and liver, and the immune organs (bursa of Fabricius, spleen, and thymus) were removed, weighed, and recorded as g/kg bodyweight. All organs were kept in 10% formalin for histopathological examination. Sacrificed birds were hygienically disposed of after sampling through burial of the carcasses, heads, shanks, feet, and viscera with the use of slaked lime beneath and above, and the area was fenced to discourage carnivorous animals.

#### Biochemical and immunological assay

Sera (a total of 315 sera samples were collected from 315 sacrificed birds: 63 birds from each experimented group) were examined for levels of total protein (expressed in g/dL), albumin (expressed in g/dL), alanine aminotransferase (expressed in IU/L), aspartate aminotransferase (expressed in IU/L), urea (expressed in mg/dL), and creatinine (expressed in mg/dL) using a Roche COBAS Integra 800 Chemistry Analyzer. Ig (IgG, IgM, and IgA; expressed in mg/dL) concentrations were measured using a Roche Elecsys 1010 Immunoassay Analyzer.

#### Bacteriological examination

Ten microliters from each of the *in vitro* microbial-probiotic mixture (256 samples, 4 replicates×4 contact times×4 cultures×4 probiotics concentrations) resuscitation tubes were dropped (using the drop plate technique as recommended by Kim and Lee [24]) onto EMB agar (Modified Levine EMB Thermo Scientific™ Oxoid™, CM0069B, 500 g) and CHROMagar (BD BBL™ CHROMagar™ *Salmonella* READY-TO-USE Plated Media) for the *E. coli* O157: H7 and *S*. Typhimurium assays, respectively, and onto SDA (Thermo Scientific™ Oxoid™ SDA, CM0041, 500 g) for the *C. albicans* and *T. mentagrophytes* assays and incubated at 37°C for 24 h.

Duodenal swabs (a total of 315 duodenal swabs were collected after sacrificing 315 birds: 63 birds from each experimented group) were prepared according to the method described by the American Public Health Association [25,26]. In brief, ten-fold serial dilutions up to 10^−8^ were prepared to screen the different opportunities for microbial growth. Bacterial counts were conducted using the drop plate technique, as recommended by Kim and Lee [24], onto standard plate count agar (SPA, Thermo Scientific™ Oxoid™ Plate Count Agar, CM0325, 500 g), EMB agar (Modified Levine Eosine Methylene Blue Thermo Scientific™ Oxoid™, CM0069B, 500 g), and CHROMagar (BD BBL™ CHROMagar™ *Salmonella* READY-TO-USE Plated Media) for the total aerobic bacterial count, total *Enterobacteriaceae* count (TEC), and total *Salmonella* count (TSC), and the plates were incubated at 37°C for 24-48 h.

Typical colonies of *E. coli*, *Salmonella*, *Candida*/*Trichophyton* on EMB agar, CHROMagar, and SDA, respectively, during the *in vitro* study were detected, counted, and compared to the original microbial counts used, and the killing rates were calculated. Total bacterial count on SPA, TEC on EMB agar, and TSC on CHROMagar cultured from the *in vivo* intestinal swabs that showed 30-300 CFU were recorded. The counting of microbial colonies during the *in vitro* and *in vivo* studies was carried using a darkfield colony counter (R164109 Reichert-Jung Quebec Darkfield 3325 Colony Counter, Fisher Scientific) [27], and the counts were converted into logarithmic numbers.

#### Histopathological examination

Tissue samples from the liver, heart, spleen, thymus, and bursa of Fabricius were washed with 5% phosphate-buffered saline (PBS) (ABI^®^ PBS, PBS, 10× concentrated, sterile, pH 7.4, without CaCl_2_ and MgCl_2_, 500 mL) and fixed through impregnation in 10% buffered formalin saline solution. The tissues were maintained until complete fixation, cut into sections of 5-mm thickness, and put into tissue cassettes. The specimens were dehydrated through transfer through a series of alcohols with different concentrations, cleared with two changes of xylol, embedded into paraffin, and cut into 4-μm-thick sections. The obtained tissue sections were stained with hematoxylin and eosin as recommended by Bancroft *et al*. [28] and Jones *et al*. [29]. Histological sections were examined using a Zeiss Axioplan microscope (Carl Zeiss MicroImaging, Thornwood, NY, USA) under 40× and photographed.

### Statistical analysis

Statistical analysis was carried out using SPSS version 20 (SPSS-20, IBM Corp., NY, USA) [30]. The recorded data were analyzed using a two-way multifactorial analysis of variance for all treated groups, age, and their interactions. Data were analyzed for the *in vitro* influence of different probiotic concentrations (0.5, 1.0*, 1.5, and 2.0 g/L) on microbial cultures after different exposure intervals (0.25, 0.5, 1, and 2 h), and for the *in vivo* influence of the probiotic concentration, broiler age, and their interactions on performance, immunity, carcass weight, immune organ weight, edible organ weights, and intestinal microbial counts. The statistical model was summarized as follow:

Y_ijk_=μ+α_i_+β_j_+(αβ)_ij_+ɛ _ijk_

Where Y_ijk_ was the measurement of the dependent variables; μ was overall mean; α_i_ was the fixed effect of the probiotic concentrations; β_j_ was the fixed effect of the broiler’s age; (αβ)_ij_ was the interaction effect of the probiotic concentrations by broiler’s age; and ɛ _ijk_ was the random error. The results were displayed in tables as high significance at p<0.01, significant at p≤0.05, and non-significant at p>0.05. The logarithmic forms (Log_10_) of total bacterial, *Enterobacteriaceae*, and *Salmonella* counts were calculated using Microsoft Excel 2016.

## Results

### *In vitro* antimicrobial actions

The *in vitro* antimicrobial actions are shown in Table-1. The overall *in vitro* antimicrobial actions of the probiotic revealed highly significant reductions (p<0.01) in *E. coli* O157: H7, *S*. Typhimurium, *C. albicans*, and *T. mentagrophytes* viable counts (up to 99.9%, 55.7%, 55.1%, and 60.2%, respectively) when exposed to 2.0 g/L *B. subtilis* suspension compared to other concentrations, regardless of the contact time. The probiotic revealed highly significant antimicrobial actions (p<0.01) as the contact time with the microbial cultures increased to 2 h.

**Table-1:** *In vitro* antimicrobial action (killing rate mean±SE) of different *Bacillus subtilis* probiotic’ concentrations at different exposure times.

Probiotic g/L	Contact times/h	Bacterial cultures (%)		Fungal cultures (%)	
	
		*Escherichia coli* O157: H7	*Salmonella* Typhimurium	*Candida albicans*	*Trichophyton mentagrophytes*
Overall means concerning probiotic concentrations					
0.5 g/L		84.3^a^±0.61	34.2^d^±0.15	52.2^a^±0.07	56.2^b^±0.8
1.0* g/L		99.2^a^±0.33	40.0^c^±0.26	52.6^a^±0.08	57.0^a, b^±0.07
1.5 g/L		99.9^a^±0.01	50.0^b^±0.33	54.0^a^±0.07	59.6^a, b^±0.09
2.0 g/L		99.9^a^±0.00	55.7^a^±0.16	55.1^a^±0.09	60.2^a^±0.06
p value		0.001	0.000	0.334	0.069
Overall means concerning contact times					
0.25 h		84.3^b^±0.31	18.5^d^±0.18	13.2^d^±0.08	18.0^d^±0.05
0.5 h		99.2^a^±0.25	27.8^c^±0.19	35.2^c^±1.19	40.0^c^±1.23
1 h		99.9^a^±0.01	54.2^b^±0.36	73.2^b^±1.65	78.0^b^±0.69
2 h		100.0^a^±0.01	79.2^a^±2.22	92.2^a^±0.53	97.0^a^±0.61
p value		0.005	0.001	0.000	0.002
Probiotics concentrations by contact times interactions					
0.5 g/L	0.25 h	40.4^c^±4.12	7.8^d^±0.71	12.0±1.63	16.0^d^±1.55
	0.5 h	97.0^b^±0.20	17.1^c^±0.00	34.0^c^±2.58	38.0^c^±1.32
	1 h	99.8^a^±0.01	43.5^b^±2.14	72.0^b^±0.65	76.0^b^±2.11
	2 h	99.9^a^±0.00	68.5^a^±1.16	91.0^a^±1.01	95.0^a^±1.02
1.0* g/L	0.25 h	97.0^b^±0.20	13.5^d^±0.66	12.4^d^±1.22	16.8^d^±0.98
	0.5 h	99.8^a^±0.01	22.8^c^±0.00	38.1^c^±0.99	39.2^c^±0.56
	1 h	100.0^a^±0.00	49.2^b^±1.98	75.8^b^±3.61	77.4^b^±1.12
	2 h	100.0^a^±0.00	74.2^a^±1.22	94.2^a^±1.55	95.3^a^±0.01
1.5 g/L	0.25 h	99.8^a^±0.01	23.5^d^±0.82	13.8^d^±1.55	19.4^d^±0.02
	0.5 h	99.9^a^±0.00	32.8^c^±0.00	39.8^c^±2.58	41.4^c^±0.52
	1 h	100.0^a^±0.00	59.2^b^±0.98	75.8^b^±3.66	79.2^b^±1.32
	2 h	100.0^a^±0.00	84.2^a^±1.32	94.8^a^±1.21	98.1^a^±1.08
2.0 g/L	0.25 h	99.9^a^±0.00	29.2^d^±1.66	14.8^d^±1.66	20.1^d^±0.62
	0.5 h	100.0^a^±0.00	38.5^c^±0.62	41.5^c^±2.55	42.0^c^±1.54
	1 h	100.0^a^±0.00	65.0^b^±1.54	79.4^b^±1.15	80.0^b^±1.54
	2 h	100.0±0.00	90.0^a^±0.02	95.6^a^±0.98	98.8^a^±1.00
p value	0.094	0.002	0.001	0.000

Means carrying different superscripts in the same column are significantly different at P≤0.05 or highly significantly different at P*<*0.01. Means carrying the same superscripts in the same column are non-significantly different at P*<*0.05. SE=Standard error,

* Recommended concentration by the manufacturer

Different contact times conferred highly significant reductions (p<0.01) in the *E. coli* O157: H7 viable count: up to 99.8% in 0.5 g/L *B. subtilis* suspension after 1 h, 100.0% in 1.0* g/L *B. subtilis* suspension after 1 h, 100.0% in 1.5 g/L *B. subtilis* suspension after 1 h, and 100.0% in 2.0 g/L *B. subtilis* suspension after 0.5 h. No significant differences were recorded between the killing efficacies recorded as a result of the exposure to 1.5 or 2.0 g/L *B. subtilis* suspension. *S*. Typhimurium also showed highly significant reductions (p<0.01): up to 68.5% in 0.5 g/L *B. subtilis* suspension after 2 h, 74.2% in 1.0* g/L *B. subtilis* suspension after 2 h, 84.2% in 1.5 g/L *B. subtilis* suspension after 2 h, and 90.0% in 2.0 g/L *B. subtilis* suspension after 2 h.

The yeast assays also revealed significant viable cell count reduction rates. *C. albicans* showed highly significant reductions (p<0.01): Up to 91.0% in 0.5 g/L *B. subtilis* suspension after 2 h, 94.2% in 1.0* g/L *B. subtilis* suspension after 2 h, 94.8% in 1.5 g/L *B. subtilis* suspension after 2 h, and 95.6% in 2.0 g/L *B. subtilis* suspension after 2 h. *T. mentagrophytes* also showed highly significant reductions (p<0.01): Up to 95.0% in 0.5 g/L *B. subtilis* solution after 2 h, 95.3% in 1.0* g/L *B. subtilis* solution after 2 h, 98.1% in 1.5 g/L *B. subtilis* suspension after 2 h, and 98.8% in 0.5 g/L *B. subtilis* suspension after 2 h.

### Growth traits

The effects of different concentrations of *B. subtilis* suspension on the growth traits of broilers are shown in Table-2. The monitoring and observations of the broilers that received probiotic treatment revealed significantly lower mortalities: 1.3% (3 out of 230 broilers) during the entire experiment. Weight gains and performance indices revealed highly significant increases (p<0.01) in broilers supplemented with 2.0, 1.5, 1.0*, and 0.5 g/L *B. subtilis* suspension (Table-2). Feed intakes showed highly significant declines (p<0.01) in broilers supplemented with 2.0, 1.5, 1.0*, and 0.5 g/L *B. subtilis* suspension. FCR revealed highly significant (p<0.01) lower and promising ratios in broilers supplemented with 2.0, 1.5, 1.0*, and 0.5 g/L *B. subtilis* suspension. Age interactions with the different treatments showed highly significant increases (p<0.01) in weight gains and performance indices at the 3^rd^, 5^th^, 4^th^, 2^nd^, and 1^st^ weeks, respectively, and highly significant increases (p<0.01) in feed intakes as age proceeded. Highly significant increases (p<0.01) were also observed in the FCR at the 5^th^, 4^th^, 3^rd^, 1^st^, and 2^nd^ weeks, respectively.

**Table-2:** Performance indices (mean±SE) in broilers supplemented with different concentrations of probiotics.

Probiotic g/L	Age/week	BWG/g	FI/g	FCR	PI
Overall means concerning probiotic concentrations					
0.5 g/L		364.6^c, d^±6.62	580.7^b^±5.95	1.58^a^±0.13	6.24^d^±0.53
1.0* g/L		383.2^b, c^±2.95	563.2^c^±5.84	1.47^b^±0.11	6.94^c^±0.62
1.5 g/L		400.8^b^±3.12	526.0^d^±5.36	1.33^c^±0.11	8.24^b^±0.72
2.0 g/L		421.9^a^±32.4	504.0^e^±5.29	1.17^d^±0.10	9.23^a^±0.82
Control		349.0^d^±2.38	589.1^a^±5.95	1.66^a^±0.13	5.71^e^±0.45
p value		0.000	0.001	0.000	0.000
Overall means concerning contact times					
1^st^ week		116.0^d^±1.84	129.1^e^±2.39	1.12^c^±0.02	1.35^e^±0.05
2^nd^ week		403.1^c^±4.16	299.8^d^±4.79	0.74^d^±0.01	7.49^d^±0.18
3^rd^ week		537.8^a^±7.74	590.4^c^±5.44	1.10^c^±0.02	10.02^a^±0.29
4^th^ week		418.0^c^±2.26	773.8^b^±5.92	2.01^b^±0.10	8.48^c^±0.68
5^th^ week		444.6^b^±1.51	969.9^a^±1.22	2.25^a^±0.07	9.02^b^±0.37
p value		0.000	0.005	0.001	0.002
Probiotics concentrations by contact times interactions					
0.5 g/L	1^st^ week	121.5^e^±1.25	140.3^e^±0.42	1.15^c^±0.01	1.32^e^±0.02
	2^nd^ week	402.3^c^±4.32	318.6^d^±0.98	0.79^d^±0.00	7.01^c^±0.11
	3^rd^ week	510.5^a^±1.73	613.0^c^±0.68	1.20^c^±0.03	8.89^a^±0.33
	4^th^ week	315.3^d^±5.05	800.6^b^±1.28	2.56^a^±0.10	5.44^d^±0.29
	5^th^ week	473.5^b^±29.96	1030.8^a^±1.35	2.22^b^±0.15	8.53^b^±0.64
1.0* g/L	1^st^ week	107.5^d^±1.36	130.8^e^±1.01	1.21^c^±0.01	1.14^e^±0.02
	2^nd^ week	415.0^b^±4.69	299.6^d^±1.54	0.72^d^±0.01	7.67^c^±0.15
	3^rd^ week	526.3^a^±7.61	601.1^c^±0.94	1.14^c^±0.01	9.46^b^±0.19
	4^th^ week	331.1^c^±1.54	789.5^b^±3.43	2.39^a^±0.07	5.92^d^±0.21
	5^th^ week	536.0^a^±2.06	994.8^a^±1.62	1.87^b^±0.07	10.5^a^±0.46
1.5 g/L	1^st^ week	103.0^c^±1.71	117.6^e^±0.88	1.14^b^±0.02	1.18^e^±0.03
	2^nd^ week	434.6^b^±4.05	285.3^d^±1.43	0.65^d^±0.00	8.67^d^±0.13
	3^rd^ week	589.4^a^±1.67	576.1^c^±1.40	0.98^c^±0.03	11.9^a^±0.52
	4^th^ week	454.2^b^±2.65	749.6^b^±1.02	1.68^b^±0.11	9.80^b^±0.62
	5^th^ week	423.0^b^±3.88	901.3^a^±0.71	2.21^a^±0.19	9.62^c^±0.99
2.0 g/L	1^st^ week	128.3^d^±2.01	111.6^e^±1.20	0.87^d^±0.02	1.84^e^±0.06
	2^nd^ week	385.0^c^±4.67	261.1^d^±2.44	0.67^e^±0.01	8.06^d^±0.21
	3^rd^ week	557.1^b^±1.30	540.6^c^±1.60	0.97^c^±0.02	11.39^b^±0.39
	4^th^ week	629.1^a^±1.24	724.1^b^±1.27	1.15^b^±0.02	14.96^a^±0.40
	5^th^ week	410.0^c^±2.12	882.6^a^±2.78	2.18^a^±0.11	9.91^c^±0.57
Control	1^st^ week	119.6^c^±1.08	145.0^e^±0.00	1.21^c^±0.01	1.25^e^±0.02
	2^nd^ week	378.8^b^±3.64	334.1^d^±2.89	0.88^d^±0.01	6.01^d^±0.09
	3^rd^ week	505.6^a^±7.07	621.1^c^±1.92	1.22^c^±0.02	8.46^a^±0.16
	4^th^ week	360.3^b^±1.52	805.1^b^±1.57	2.25^b^±0.09	6.29^c^±0.30
	5^th^ week	380.8^b^±1.94	1040.0^a^±3.41	2.76^a^±0.13	6.54^b^±0.38
p value		0.000	0.001	0.000	0.001

Means carrying different superscripts in the same column are significantly different at (p≤0.05) or highly significantly different at (p<0.01). Means carrying the same superscripts in the same column are non significantly different at P*<*0.05. LBW=Live body weight, BWG=Body weight gain, FCR=Feed conversion ratio, PI=Performance index, SE=Standard error,

* Recommended concentration by the manufacturer

### Live, carcass, and immune organ weights

The effects of different concentrations of *B. subtilis* suspension on the live, carcass, and immune organ weights of broilers are shown in Table-3. Live body, carcasses, immune organs (bursa of Fabricius and thymus), and edible organs (liver and heart) weights, revealed highly significant increases (p<0.01) in the broilers supplemented with 2.0 g/L *B. subtilis* suspension compared to other supplemented groups and the unsupplemented control group, with no significant differences between heart weights of the broilers supplemented with 2.0 and 1.5 g/L *B. subtilis*. On the other hand, spleen’s weights revealed highly significant declines (p<0.01) in all treated broilers compared to the unsupplemented control group.

**Table-3:** Live body, carcass, and immune organs’ weight (mean±SE/g) in broilers supplemented with different concentrations of probiotics.

Probiotic g/L	LBW/g	Carcass weight/g	Edible organs weights/g		Immune organs weights/g		
	
			Liver/g	Heart/g	Bursa/g	Spleen/g	Thymus/g
Overall means concerning probiotic concentrations							
0.5 g/L	1876^d^±4.63	1555^d^±4.6	20.5^d^±0.18	10.3^d^±0.02	1.45^e^±0.01	1.51^e^±0.04	2.51^b, c^±0.08
1.0* g/L	1923^c^±6.85	1692^c^±6.8	21.7^c^±0.22	11.5^c^±0.09	1.78^d^±0.01	1.74^c^±0.06	2.10^d^±0.03
1.5 g/L	2102^b^±14.39	1892^b^±14.3	23.1^b^±0.16	12.9^b^±0.05	1.86^c^±0.04	1.61^d^±0.01	2.45^c^±0.02
2.0 g/L	2191^a^±12.00	1996^a^±12.0	24.8^a^±0.16	14.5^a^±0.03	2.33^a^±0.05	2.46^b^±0.07	2.97^a^±0.06
Control	1789^e^±6.21	1433^e^±6.2	19.2^e^±0.09	9.2^e^±0.02	2.12^b^±0.05	2.62^a^±0.01	2.56^b^±0.03
p value	0.000	0.000	0.000	0.003	0.001	0.001	0.001

Means carrying different superscripts in the same column are significantly different at P≤0.05 or highly significantly different at P*<*0.01. Means carrying the same superscripts in the same column are non-significantly different at P>0.05. LBW=Live body weight, SE=Standard error,

* Recommended concentration by the manufacturer

### Intestinal microbial load and immunoglobulin concentration

The effects of different concentrations of *B. subtilis* suspension on the intestinal microbial load and immunoglobulin concentration of broilers are shown in Table-4. The total bacterial counts revealed highly significant declines (p<0.01) in broilers supplemented with 2.0 g/L *B. subtilis*, with no significant differences between broilers supplemented with 2.0 and 1.5 g/L *B. subtilis*. Meanwhile, the total *Enterobacteriaceae* and *Salmonella* counts showed highly significant declines (p<0.01) in broilers supplemented 2.0 g/L *B. subtilis* compared with the other supplemented groups and the control group.

**Table-4:** Logarithmic bacterial counts (mean±SE CFU/mL) and Immunoglobulin concentrations (mean±SE mg/dL) in broilers supplemented with different concentrations of probiotics.

Probiotic g/L	Log. bacterial counts			Immunoglobulin concentrations		
	
	Log TBC CFU/mL	Log TEC CFU/mL	Log TSC CFU/mL	IgG mg/dL	IgM mg/dL	IgA mg/dL
Overall means concerning probiotic concentrations						
0.5 g/L	4.20^b^±0.01	3.50^a^±0.05	2.40^a^±0.01	1359^d^±0.01	254^d^±0.00	143^c^±0.01
1.0* g/L	4.14^c^±0.00	3.45^b^±0.01	2.32^b^±0.00	1495^c^±0.02	300^c^±0.00	158^b^±0.01
1.5 g/L	4.06^d^±0.02	3.42^c^±0.03	1.43^c^±0.00	1540^b^±0.02	406^b^±0.01	155^b^±0.01
2.0 g/L	4.06^d^±0.02	2.70^d^±0.04	0.60^d^±0.01	1794^a^±0.01	499^a^±0.00	243^a^±0.02
Control	4.74^a^±0.01	2.49^e^±0.03	0.47^e^±0.01	1318^d^±0.02	204^e^±0.01	153^b^±0.01
p value	0.001	0.001	0.001	0.000	0.000	0.001

Means carrying different superscripts in the same column are significantly different at P≤0.05 or highly significantly different at P*<*0.01. Means carrying the same superscripts in the same column are non-significantly different at P*<*0.05. TBC=Total bacterial count, TEC=Total Enterobacteriaceae count, TSC=Total Salmonella count, IgG=Immunoglobulin G, IgM=Immunoglobulin M, IgA=Immunoglobulin A, Log=Logarithm, SE=Standard error,

* Recommended concentration by the manufacturer

Immunoglobulin IgG, IgM, and IgA concentrations were found to be significantly increased (p<0.01) in broilers supplemented with 2.0 g/L *B. subtilis* compared to the other supplemented groups and the control group.

### Biochemical profile

The effects of different concentrations of *B. subtilis* suspension on the biochemical profiles of broilers are shown in Table-5. Total protein, albumin, alanine aminotransferase, and aspartate aminotransferase levels were found to be significantly improved (p<0.01) in broilers supplemented with 2.0 g/L *B. subtilis* compared to the other treated groups and the untreated controls. Meanwhile, urea and creatinine levels showed a significant decline (p<0.01) in broilers supplemented with 2.0 g *B. subtilis*/L drinking water compared to the other supplemented groups and the control group.

**Table-5:** Biochemical parameters (mean±SE) in broilers supplemented with different concentrations of probiotics.

Probiotic g/L	TP g/dL	ALB g/dL	ALT IU/L	AST IU/L	Urea mg/dL	Creat mg/dL
Overall means concerning probiotic concentrations						
0.5 g/L	3.57^d^±0.00	1.87^d^±0.00	27.1^b^±0.02	36.1^b^±0.02	26.4^b^±0.23	0.56^c^±0.01
1.0* g/L	4.39^b^±0.02	3.17^b^±0.00	26.9^c^±0.05	36.1^b^±0.09	25.2^c^±0.13	0.74^a^±0.01
1.5 g/L	4.39^b^±0.01	2.37^c^±0.01	25.7^d^±0.08	34.8^c^±0.13	25.0^c^±0.05	0.49^d^±0.02
2.0 g/L	4.99^a^±0.01	3.87^a^±0.01	27.4^a^±0.23	36.6^a^±0.07	24.3^d^±0.33	0.44^e^±0.01
Control	3.59^c^±0.01	1.87^d^±0.00	27.2^b^±0.08	36.1^b^±0.10	27.2^a^±0.13	0.59^b^±0.01
p value	0.005	0.001	0.000	0.006	0.000	0.000

Means carrying different superscripts in the same column are significantly different at P≤0.05 or highly significantly different at P*<*0.01. Means carrying the same superscripts in the same column are non-significantly different at P>0.05. TP=Total protein, ALB=Albumin, ALT=Alanine aminotransferase, AST=Aspartate aminotransferase, Urea=Urea, Creat=Creatinine, SE=Standard error,

* Recommended concentration by the manufacturer

### Histopathological examination

**Figure-1:**
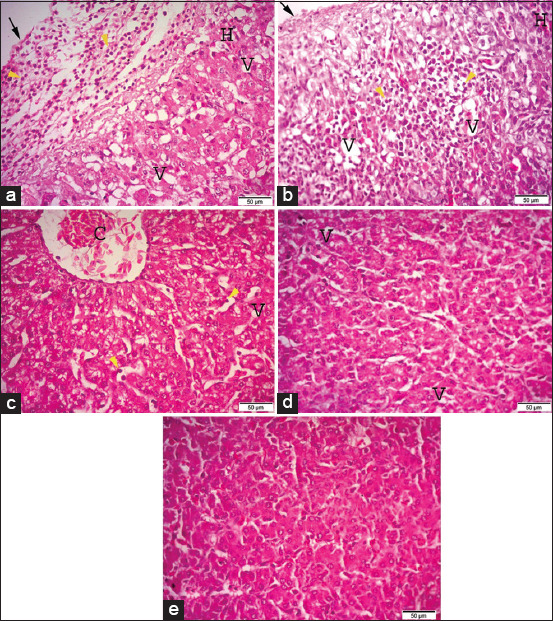
Photomicrographs of hematoxylin and eosin (H&E) stained sections of the liver, (a) the liver of G1 (broilers supplemented with 0.5 g/L *Bacillus subtilis*) showing adhesive perihepatitis (arrow), mononuclear cell infiltration (arrowhead), vaculation of hepatocytes cytoplasm (V) with mild hemorrhage (H). (b) The liver of G2 (broilers supplemented with 1.0* g/L *B. subtilis*). (c) The liver of G3 (broilers supplemented with 1.5 g/L *B. subtilis*). (d) The liver of G4 (2.0 g/L *B. subtilis*). (e) The liver of G5 (control group). H&E (40×). Bar 50 μm.

**Figure-2:**
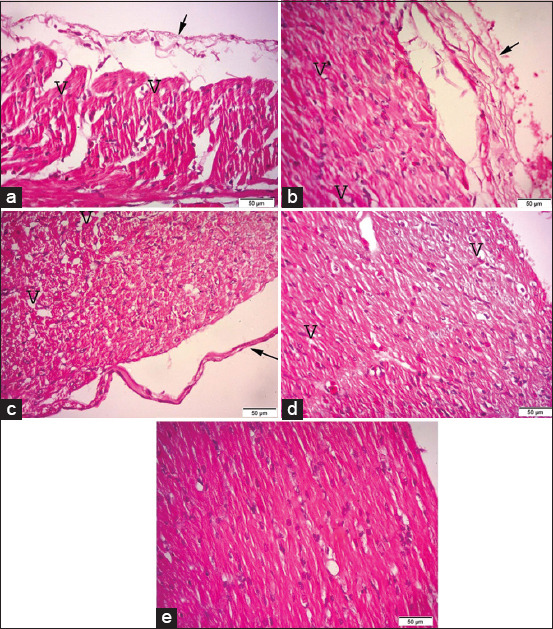
Photomicrographs of hematoxylin and eosin (H&E) stained sections of the heart, (a) the heart of G1 (broilers supplemented with 0.5 g/L *Bacillus subtilis*) showing pericarditis (arrow), vaculation of myocardial cells (V). (b) The heart of G2 (broilers supplemented with 1.0* g/L *B. subtilis*). (c) The heart of G3 (broilers supplemented with 1.5 g/L *B. subtilis*). (d) The heart of G4 (broilers supplemented with 2.0 g/L *B. subtilis*). (e) The heart of G5 (control group). H&E (40×). Bar 50 μm.

**Figure-3:**
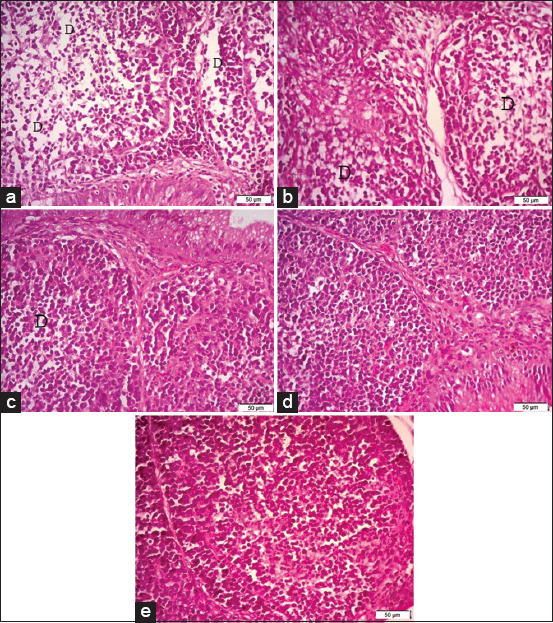
Photomicrographs of hematoxylin and eosin (H&E) stained sections of the bursa of Fabricius, (a) the bursa of Fabricius of G1 (broiler supplemented with 0.5 g/L *Bacillus subtilis*) showing severe depletion of lymphoid follicles (D). (b) The bursa of Fabricius of G2 (broiler supplemented with 1.0* g/L *B. subtilis*). (c) The bursa of Fabricius of G3 (broiler supplemented with 1.5 g/L *B. subtilis*). (d) The bursa of Fabricius of G4 (broiler supplemented with 2.0 g/L *B. subtilis*). (e) The bursa of G5 (control group). H&E. (40×). Bar 50 μm.

**Figure-4:**
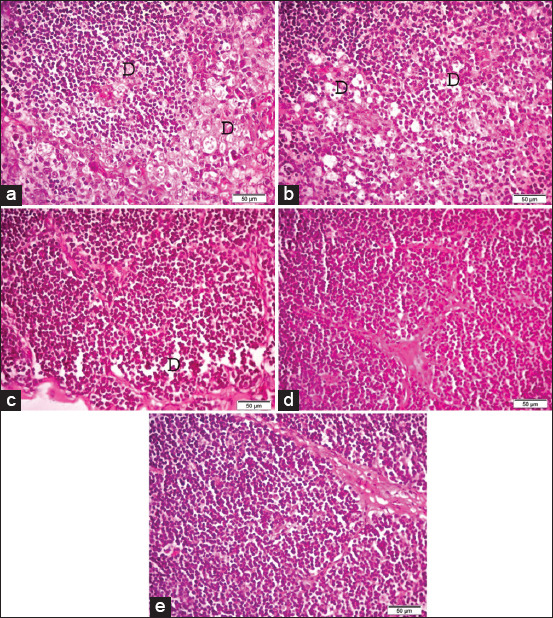
Photomicrographs of hematoxylin and eosin (H&E) stained sections of the thymus (a) the thymus of G1 (broilers supplemented with 0.5 g/L *Bacillus subtilis*) showing severe depletion of lymphoid follicles (D). (b) The thymus of G2 (broilers supplemented with 1.0* g/L *B. subtilis*). (c) The thymus of G3 (broilers supplemented with 1.5 g/L *B. subtilis*). (d) The thymus of G4 (broilers supplemented with 2.0 g/L *B. subtilis*). (e) The thymus of G5 (control group). H&E. (40×). Bar 50 μm.

**Figure-5:**
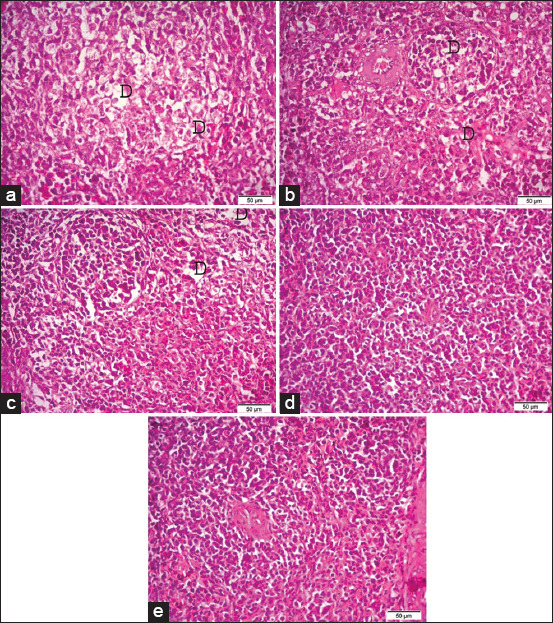
Photomicrographs of hematoxylin and eosin (H&E) stained sections of the spleen (a) the spleen of G1 (broilers supplemented with 0.5 g/L *Bacillus subtilis*) showing severe depletion of lymphoid follicles (D). (b) The spleen of G2 (broilers supplemented with 1.0* g/L *B. subtilis*). (c) The spleen of G3 (broilers supplemented with 1.5 g/L *B. subtilis*). (d) The spleen of G4 (broilers supplemented with 2.0 g/L *B. subtilis*). (e) The spleen of G5 (control group). H&E (40×). Bar 50 μm.

The results of the histopathological examination after supplementation with different concentrations of *B. subtilis* suspension are shown in Figures-1-5. The photomicrographs of the livers of broilers supplemented with 0.5 g/L (Figure-1a) and 1.0* g/L (Figure-1b) *B. subtilis* suspensions reveal adhesive perihepatitis with severe leukocytic infiltrations, and hepatocyte examination revealed severe vacuolation of the cytoplasm with mild areas of hemorrhage compared to control (Figure-1e). Broilers supplemented with 1.5 g/L *B. subtilis* suspension (Figure-1c) showed vacuolated cytoplasm in the liver, congestion of the central vein, and mild leukocytic infiltrations compared to the controls (Figure-1e). Meanwhile, the livers of broilers supplemented with 2.0 g/L *B. subtilis* (Figure-1d) showed pronounced improvement of the histopathological picture.

The histopathological examinations of the heart (Figures-2a and b) revealed severe fibrinous pericarditis with degeneration of the myocardium, and some of the myocardial cells showed vacuolated cytoplasm with leukocytic infiltrations in broilers supplemented with 0.5 and 1.0* g/L *B. subtilis*, respectively, compared to the controls (Figure-2e). The hearts of the broilers supplemented with 1.5 g/L *B. subtilis* (Figure-2c) showed mild pericarditis with mild myocarditis and vacuolated cytoplasm in some of the myocardial cells. The hearts of the broilers supplemented with 2.0 g/L *B. subtilis* (Figure-2d) showed an improved and near-normal histopathological picture.

Stained histopathological sections of the bursas of Fabricius (Figures-3a and b), thymuses (Figures-4a and b), and spleens (Figures-5a and b) of the broilers supplemented with 0.5 and 1.0* g/L *B. subtilis*, respectively, revealed severe lymphoid depletion compared to the controls (bursa of Fabricius: Figure-3e, thymus: Figure-4e, spleen: Figure-5e). Broilers supplemented with 1.5 g/L *B. subtilis* showed mild lymphoid depletion in the bursa of Fabricius (Figure-3c), thymus (Figure-4c), and spleen (Figure-5c). The bursas of Fabricius, thymuses, and spleens of the broilers that were supplemented with 2.0 g/L *B. subtilis* showed lymphoid hyperplasia (Figures-3d-5d) compared to the normal view of the bursa of Fabricius in the controls (Figure-3e, thymus in Figure-4e, and spleen in Figure-5e).

## Discussion

Probiotics are effective and promising feed and water additives in the field of preventive measures and therapeutics for broiler chickens [31,32]. Probiotics can enhance and improve the intestinal mucosa and microbiota, thus improving the performance and production of broiler chickens [33,34]. *B. subtilis* has been categorized as a type of probiotic bacteria that naturally inhabit the intestine of healthy broilers and can promote gut conditions [35,36], absorption functions, performance [37,38], and immunity [39], and can alleviate many overwhelming challenges, such as microbial stress and heat stress [40].

The current *in vitro* results revealed that the *B. subtilis* 2.0 g/L suspension was able to produce direct significant *in vitro* antimicrobial action against *E. coli* O157: H7 (4.5×10^12^ CFU/mL) and *S*. Typhimurium (1.2×10^7^ CFU/mL) after 0.25 and 2 h, respectively. Supplementing broilers with 1.5 or 2.0 g *B. subtilis* (5×10^6^ CFU/g/L of drinking water) induced significant reductions in total bacterial, *Enterobacteriaceae*, and *Salmonella* counts. These *in vitro* and *in vivo* antimicrobial actions may be attributable to direct-fed microbes, organic acid production, and protein killing molecules released from *B. subtilis*. The current results were consistent with those in the study by Oh *et al*. [41], who found that supplementing broilers with probiotics reduced coliform and *E. coli* counts in *Salmonella-*challenged broilers. Ebrahimi *et al*. [42] concluded that acidic pH from organic acids produced by supplementing broilers with PrimaLac^®^ (120 g/1 L water until the 14^th^ day, 454 g/1000 kg ration until the 28^th^ day, and 225 g/1000 kg ration for the rest of the growing period) reduced growth and colonization of *Campylobacter jejuni*. Nishiyama *et al*. [43] and Saint-Cyr *et al*. [44] reported that using *Lactobacillus gasseri* SBT2055 and *Bacillus* sp. suppressed the growth and colonization of *C. jejuni*. Carter *et al*. [45] reported similar results when they used a mixture of *Enterococcus faecium* and *Lactobacillus salivarius* to inhibit the growth and colonization of *S*. Enteritidis. Neveling *et al*. [46] revealed that multispecies probiotics composed of *Lactobacillus crispatus*, *L. salivarius*, *Lactobacillus gallinarum*, *Lactobacillus johnsonii*, *Enterococcus faecalis*, and *Bacillus amyloliquefaciens* could inhibit the colonization of *S*. Enteritidis in the intestine when administered to broiler chickens.

The current results showed that the *B. subtilis* 2.0 g/L suspension was able to produce significant *in vitro* antimicrobial action against *C. albicans* (2.5×10^6^ CFU/mL) and *T. mentagrophytes* (2.5×10^6^ CFU/mL) after 2 h. The current results correlate well with those of Zhang *et al*. [47], who reported that *B. subtilis* ANSB060 could neutralize the aflatoxins produced by *Aspergillus pseudotamarii*, *Aspergillus flavus*, *Aspergillus niger*, *Aspergillus parasiticus*, *Aspergillus ochraceoroseus*, and *Aspergillus nomius* and, when administered to broilers, could improve growth and increase weight gains. Fan *et al*. [48] found that the inclusion of *B. subtilis* in the diets of broilers could improve the intestinal microbiota and neutralize the harmful influence of aflatoxin-producing agents by degrading the cell wall polysaccharide contents of aflatoxin-producing agents. Abdolmaleki *et al*. [49] reported that probiotic *Bacillus* spp. MBIA2.40 had a protective influence. They found that the inclusion of *Bacillus* spp. MBIA2.40 in diets contaminated with fungal growth and their associated aflatoxins could minimize the negative influences of these fungal organisms and their toxins in broiler chickens.

Probiotics can exert their action through various methods, including blocking bacterial binding sites (competitive inhibition), regenerating intestinal mucosa, and enhancing the secretion of digestive enzymes. In our study, supplementing broilers with 1.5 and 2.0 g *B. subtilis* (5×10^6^ CFU/g in each liter of drinking water) enhanced the live body weight, accelerated weight gains, and improved performance indices compared to 1.0* g *B. subtilis* (the quantity recommended by the manufacturer). The observed actions could be attributed to the enhancing influence of *B. subtilis* probiotics on intestinal permeability and absorption functions. The results were consistent with those of He *et al*. [50] and Hosseini *et al*. [51], who reported that the inclusion of probiotics in broiler chicken rations could improve nutrient digestion, performance, antioxidant levels and activity, and intestinal morphology barriers against pathogenic microorganisms. Sobczak and Kozłowski [52] recorded synchronized results and found that *B. subtilis* (1×10^8^ CFU/kg feed) improved performance, egg quality, and yolk cholesterol contents. Kim and Lillehoi [53] concluded in their study that probiotics can be beneficial for the growth and performance of broiler chickens and could enhance disease resistance. Ribeiro *et al*. [54], Buta *et al*. [55], Meyer *et al*. [56], and Dong *et al*. [57] found that *B. subtilis* supplementation could alter the intestinal permeability and enhance the productive performances of broiler chickens.

In our study, the broilers that were supplemented with 1.5 and 2.0 g *B. subtilis* (5×10^6^ CFU/g in each liter of drinking water) showed increased carcass weights and increased edible and immune organ weights. The results were supported by those of Hrnčár *et al*. [58], who used a 20-g *Bacillus amyloliquefaciens*/kg ration for 35 days and found enhanced performance, carcass quality, and improved digestibility. Hassan *et al*. [59] concluded that using *Bacillus* sp., *Clostridium butyricum* probiotics (0.05% Saltose, 0.05% Clostat, and 0.05% Clostridium-stop), and phytobiotics (0.1% Sangrovit) significantly improved carcass traits compared to antibiotics (0.025% bacitracin methylene di-salicylate). Javandel *et al*. [60] investigated the influence of powdered *Heracleum persicum* and probiotic combinations on the performance and carcass quality of 270 1-day-old broilers and revealed significantly higher body weight gains, body weights, and carcass weights, with significantly lower abdominal fat.

Probiotics can produce immune-stimulant activity by impacting T and B effector cells, T cell regulators, enterocytes, and antigen-presenting cells, as reported by Alagawany *et al*. [61]. The current study revealed an immune-stimulant influence of probiotics in all tested concentrations, but the most prominent increase was recorded in the IgG, IgM, and IgA concentrations of the broilers supplemented with 2.0 g *B. subtilis* (5×10^6^ CFU/g/L of drinking water). The results were consistent with those reported by Harimurti and Ariyadi [62], who found a great ability of probiotics to stimulate Peyer’s patches activities, plasma cell functions, and immunoglobulin secretions. They also reported increased expression of claudin-1, -3, and -5 mRNA in broilers supplemented with probiotics.

Trani *et al*. [63] reported the abilities of probiotics to enhance gut mucosal immunity by increasing the levels of secretory IgA. Yisa *et al*. [64] and Awais *et al*. [65] concluded that the inclusion of 1 g of probiotics in the diet can be sufficient for stimulating the immune system and allowed the proliferation of beneficial microorganisms in the gut. Ashraf and Shah [66] reported that probiotics could enhance gut mucosal immunity by increasing the levels of IgA. Gonmei *et al*. [67] concluded that *Lactobacillus reuteri* PIA16, previously isolated from the chicken gut, could enhance the humoral and cell-mediated immunity of broiler chickens, and lower the mortality and susceptibility to disease. Sarwar *et al*. [68] showed that probiotics, when administered with vaccines, at a rate of 2.0 g/500 mL of water, could induce an improvement in antibody titer.

Broilers supplemented with 1.5-2.0 g *B. subtilis* (5×10^6^ CFU/g/L of drinking water) in our study showed significant increases in total protein, albumin, alanine aminotransferase, and aspartate aminotransferase and significant declines in urea and creatinine. The enhancing influence of probiotic *B. subtilis* on the biochemical profile could be attributable to the regulation of gastrointestinal permeability and enhanced physiological function. The results were compatible with those of Shankar *et al*. [69], who recorded improved serum levels of total protein, albumin, and high-density lipoproteins and significantly lower serum levels of total cholesterol and low-density lipoproteins in broilers supplemented with 0.1, 0.15, and 0.2% *Saccharomyces cerevisiae*. Deraz [70] recorded improved levels of total protein and glucose in broilers supplemented with *Lactococcus lactis* at a rate of 10^9^ CFU/mL and *Lactobacillus plantarum* at a rate of 10^12^ CFU/mL. The current results were consistent with those of Hussein and Selim [71], who investigated the efficiency of 0.5% dried yeast (S. cerevisiae), 0.5% multi-strain probiotics (*Lactobacillus acidophilus*, *B. subtilis*, and *Aspergillus oryzae*), and 0.25% dried yeast and multi-strain probiotics in broilers. They found higher total protein, globulin, and glucose levels in all of the treatments compared to the controls. Hussein *et al*. [72] recorded significantly lowered levels of alanine aminotransferase and glucose in broilers that were supplemented with *B. subtilis* probiotics.

The histopathological photomicrographs revealed that supplementing broilers with 2.0 g/L *B. subtilis* probiotics could produce improved and near-normal histopathological pictures of livers and hearts with lymphoid hyperplasia in the bursas of Fabricius, thymuses, and spleens of broilers challenged with *E. coli* O157: H7 and *S*. Typhimurium. The current results were consistent with those reported by Abramowicz *et al*. [73] and Adhikari *et al*. [74], who revealed that *B. subtilis* supplementation in broiler chickens was able to enhance the histopathological structure of the intestine, improved intestinal microbiota actions, and enhanced its barriers to reduce the incidence of necrosis. Huang *et al*. [75] also revealed that supplementing broiler chickens with *C. butyricum* probiotics improved the structure of the intestinal walls, reduced the incidence of necrotic enteritis, and enhanced the local immune response against pathogenic microorganisms. Olnood *et al*. [76] found that feeding broiler chickens on *B. subtilis* induced significant increases in total villus area and villus length and minimized the incidence of pathogenic lesions that arose from the challenge with *C. perfringens*. They also recorded near-normal liver histopathological architecture with mild lymphocytic infiltrations among hepatocytes, while in the intestine they recorded normal intestinal villi with mild metaplasia of the columnar epithelium lining the villi into goblet cells. Kogut [77] recommended probiotics in broiler chickens for their modulatory actions on the intestinal microbiota and their ability to enhance the histopathological picture of the intestine.

## Conclusion

*B. subtilis* probiotic supplementation at a concentration of 5×10^6^ CFU/g and a rate of 1.5-2.0 g/L of drinking water produced a suitable intrinsic environment for enhancing and flourishing the commensal intestinal microbiota, improved the absorption activity, increased weight gains, enhanced the performance, increased the carcass weights, and improved the biochemical parameters in broiler chickens. The supplementation also initiated immune-stimulating action by increasing immunoglobulin concentrations (IgG, IgM, and IgA). *B. subtilis* probiotics produced significant *in vitro* antimicrobial action against *E. coli* O157: H7 (4.5×10^12^ CFU/mL), *S*. Typhimurium (1.2×10^7^ CFU/mL), *C. albicans* (2.5×10^6^ CFU/mL), and *T. mentagrophytes* (2.5×10^6^ CFU/mL), and minimized and/or prevented the colonization of *E. coli* O157: H7 and *S*. Typhimurium in 14-day-old broiler chickens overwhelmed with *E. coli* O157: H7 (4.5×10^12^) and *S*. Typhimurium (1.2×10^7^ CFU/mL).

## Authors’ Contributions

ESS designed the *in vitro* and *in vivo* experimental design, participated, and supervised the execution of the experiment, and contributed to the writing of the manuscript. RTH conducted the histopathological examinations and contributed to the writing of the manuscript. MSA participated in the execution of the experiment and contributed to the writing of the manuscript. All authors have read and approved the final manuscript.
